# Metabolite Identification through Machine Learning — Tackling CASMI Challenge Using FingerID

**DOI:** 10.3390/metabo3020484

**Published:** 2013-06-06

**Authors:** Huibin Shen, Nicola Zamboni, Markus Heinonen, Juho Rousu

**Affiliations:** 1 Helsinki Institute for Information Technology HIIT; Department of Information and Computer Science, Aalto University, Konemiehentie 2, FI-02150 Espoo, Finland; E-Mail: juho.rousu@aalto.fi; 2 Institute of Molecular Systems Biology, ETH Zürich, Wolfgang-Pauli Street 16, 8093 Zürich, Switzerland; E-Mail: zamboni@imsb.biol.ethz.ch; 3 IBISC, Université d’Evry-Val d’Essonne, Bâtiment IBGBI, 23 Bd de France, 91037 cedex Evry, France; E-Mail: markus.heinonen@ibisc.fr

**Keywords:** metabolite identification, molecular fingerprints, machine learning, FingerID

## Abstract

Metabolite identification is a major bottleneck in metabolomics due to the number and diversity of the molecules. To alleviate this bottleneck, computational methods and tools that reliably filter the set of candidates are needed for further analysis by human experts. Recent efforts in assembling large public mass spectral databases such as MassBank have opened the door for developing a new genre of metabolite identification methods that rely on machine learning as the primary vehicle for identification. In this paper we describe the machine learning approach used in FingerID, its application to the CASMI challenges and some results that were not part of our challenge submission. In short, FingerID learns to predict molecular fingerprints from a large collection of MS/MS spectra, and uses the predicted fingerprints to retrieve and rank candidate molecules from a given large molecular database. Furthermore, we introduce a web server for FingerID, which was applied for the first time to the CASMI challenges. The challenge results show that the new machine learning framework produces competitive results on those challenge molecules that were found within the relatively restricted KEGG compound database. Additional experiments on the PubChem database confirm the feasibility of the approach even on a much larger database, although room for improvement still remains.

## 1. Introduction

Metabolomics is the science of measuring and analyzing the pool sizes of metabolites, which collectively define the metabolome of a biological sample [1]. Metabolomics has numerous and diverse applications in medicine, pharmaceutical research, nutrition, forensics, anti-doping, plant research and biotechnology. Due to its unparalleled sensitivity and selectivity, mass spectrometry (MS) is a cornerstone measurement technology in metabolomics.

Identification of metabolites from mass spectra is a prerequisite for further biological interpretation of metabolomics samples and metabolic modeling [1,2]. It is also the most time-consuming and laborious step in a metabolomics experiment [3].

An MS measurement of a metabolomics sample results in a set of peaks representing the mass-to-charge (m/z) ratios and intensities of the different metabolites of the sample. The fact that the same elemental composition and the same mass-to-charge ratio can arise from various different structures, as well as noise, adducts and fragments, hampers the identification of metabolites from MS data [4].

Tandem mass spectrometry (MS/MS) facilitates metabolite identification by fragmenting the detected compound and measuring the m/z ratios of the resulting fragment ions. Querying measurement spectra against spectral reference databases [5] and manual curation by domain experts dominate the current approaches via MS/MS.

The reference database method is reliable as long as the database contains the corresponding spectrum, and the query and reference spectra are measured with compatible, or ideally, identical mass spectrometers with closely matching operating parameters. Due to the general similarity of spectra, misleading false positives can occur even if the database does not contain the correct spectrum. Indeed, conventional methods are often only able to identify a minority of the detected compounds, as low as 10% [6] to 30% [7]. Unsurprisingly, a recent survey posed to MS experts found metabolite identification as the most important bottleneck in metabolomics today [3].

To alleviate the shortcomings of the reference database methods, computational approaches to model the fragmentation processes have been undertaken. Current state-of-the-art methods are based on combinatorial algorithms and database searches. Computation of fragmentation trees is tackled with several approaches [3,8,9,10,11]. The MetFrag software filters the compound databases by the precursor mass of the query mass spectra and for every candidates after filtering, the fragmentations are simulated and compared with the observed peak list [10]. SIRIUS used the analysis of isotopic patterns to give additional data on the metabolite’s elemental composition [11].

Fueled by public mass spectral databases such as MassBank [5], the use of machine learning now represents a promising and so far under-utilized approach to improve the accuracy of metabolite identification and to decrease the burden of manual tuning of metabolite identification methods. A machine learning approach for metabolite identification through molecular fingerprints was very recently introduced [12]. FingerID [13] relies on a two-step scheme. Instead of directly learning a mapping from the spectrum to the metabolite, a set of characterizing fingerprints of the metabolite is first predicted from its tandem mass spectrum using a kernel-based approach. The fingerprint prediction model is learned from a large set of tandem mass spectra obtained from the public mass spectral database MassBank [5]. In the next step, the predicted fingerprints are matched against a molecular database such as KEGG [14] or PubChem [15] to obtain a list of candidate metabolites. The metabolite identification model is thus generalized to metabolites that are not presented in reference spectral databases. Due to the machine learning approach, data from any type of mass spectrometer is supported.

In this paper, approaches used in the Critical Assessment of Small Molecule Identification (CASMI) [16] challenge, the first small molecule identification challenge in computational mass spectrometry community, are explained. In Section 2, the main methods underlying the FingerID framework [12] and the new user interfaces are presented. In Section 3 the experimental data and methods are described followed by results in Section 4. Section 5 concludes the article with future work.

## 2. Metabolite Identification through FingerID

**Figure 1:**
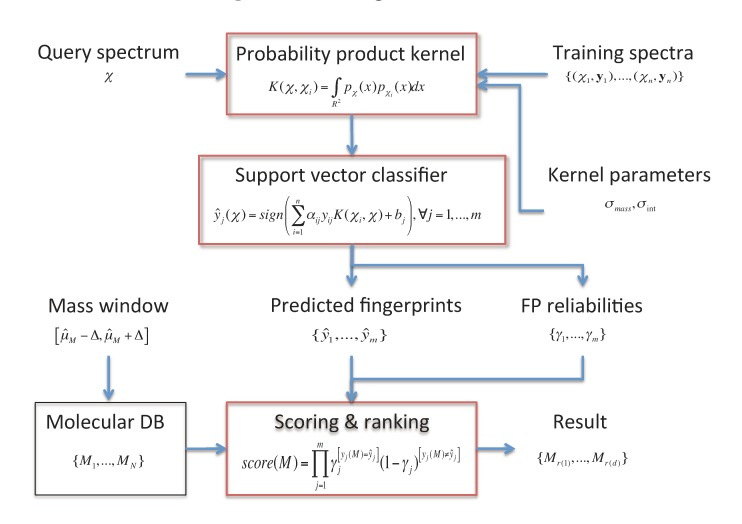
The FingerID framework.

In this section, the FingerID metabolite identification framework [12] is described. An overview of the framework is shown in Figure 1. It consists of two main modules:

- A molecular fingerprint prediction module that relies on support vector machines (SVM) equipped with a probability product kernel representation of mass spectra.
- A molecule scoring and ranking module uses the predicted fingerprints to retrieve the best matching candidate molecules from a molecular database such as KEGG or PubChem.

We describe these modules in the following. In addition, we outline the web server running FingerID as well as the software distribution package available for download.

### 2.1. Fingerprint Prediction through SVM

We define a mass spectrum *χ* = {*χ*(1),...,*χ*(*l_χ_*)} as a set of *l_χ_* peaks *χ*(*k*) = (*µ*(*k*), *ι*(*k*)) ∈ 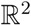, (*k* = 1,...,*l_χ_*) consisting of the peak mass *µ*(*k*) and the normalized peak intensity *ι*(*k*). Our goal is to learn a mapping between the mass spectra *χ* ∈ 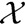 and a set of m molecular fingerprints 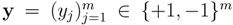. The fingerprints encode molecular properties with the value *y_j_* = +1 denoting the presence of *j^th^* property in the corresponding molecule.

We estimate the mapping *f* : 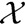 →{+1, −1}*^m^* using support vector machines (SVM) [17]. For each fingerprint, a separate SVM model

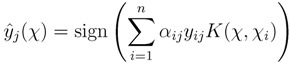
(1)
was built using a training set {(*χ*_1_, *y*_1_),...,(*χ_n_*, *y_n_*)} of tandem mass spectra of metabolites with their associated fingerprints. In Equation (1), the coefficients *α_ij _* > 0 are dual variables denoting support vectors, training examples *χ_i_* that have margin less or equal to unity in the model of the *j^th^* fingerprint. The kernel *K* measures the pairwise similarities of the spectra (see next subsection), and *y_ij_* denotes the presence or absence of the *j^th^* fingerprint in the *i^th^* training molecule.

### 2.2. Probability Product Kernel

Previously, two types of kernels were proposed [12] for mass spectra, *i.e.*, integral mass kernel and probability product kernel [18]. Integral kernel bins the mass of the peak to the nearest integer and takes it as the index in the feature vector. Integral mass kernel has a simple intuitive interpretation, however, it ignores the accurate mass of the peak and treats all peaks within [*µ* − 0.5, *µ* + 0.5] as identical to mass *µ*. On the other hand, the probability product kernel assumes that the observed peak mass and intensity is only an approximation to the true mass and intensity by placing a 2-D Gaussian distribution over the observed peaks and intensities with Gaussian noise reflecting measurement errors. In practice, the probability product kernel achieves consistently better results [12]. Hence, in this paper and the CASMI challenges, the probability product kernel was used.

The peaks *χ*(*k*) of mass spectrum *χ* are represented with Gaussian distributions 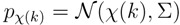 centered around the peak measurement and with covariance shared with all peaks

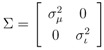



The variances 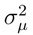 and 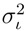 for the mass and the intensity, respectively, are both estimated from data and no covariance is assumed between them. The spectrum *χ* is finally represented as a mixture of its peak distributions 
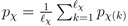
.

The probability product kernel *Kpeaks* between the peaks of two spectra *χ*, *χ'* is now given by an all-against-all matching of the underlying peak distributions [12]:

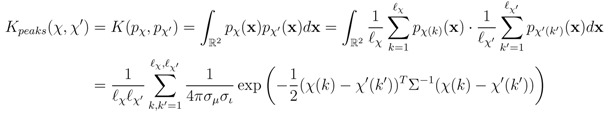
(2)


The kernel is computationally efficient as the integrals take a closed form that can be solved analytically without numerical integration.

Two additional variants of the probability product kernel were considered, which differ in the way of deriving the underlying probability distributions:

- Mass loss kernel *K_mloss_* records the difference between a fragment peak and the theoretical precursor peak by centering a Gaussian at the difference, giving the probability

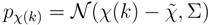

where *χ̃* = (*µ_prec_*, *ι*(*k*)) is a dummy peak with the precursor mass and the same intensity as the peak *χ*(*k*). This kernel can be interpreted as capturing putative cleaved fragments or combinations of them.
- Mass difference kernel *K*_*diff*_ computes the difference of all pairs of peaks and centers the Gaussian at the peak difference 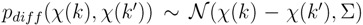. This kernel can be seen as a generalization of the mass loss kernel by not fixing a precursor mass but instead recording all possible fragmentation reactions between the peaks of two mass spectra. The kernel computation has quadratically higher complexity compared with the other two variants.

The above base kernels can be combined to several types of spectral features. The experiments in [12] showed that the combination of the *peaks* and *mloss* kernels demonstrates a good prediction accuracy and shorter computation time compared with combinations involving *K*_*diff*_. The *K*_*peaks*+*mloss*_ was used in the CASMI challenge.

### 2.3. Candidate Retrieval

Given the predicted fingerprint vector corresponding to a tandem mass spectrum, the candidate molecules matching these fingerprints are retrieved from a molecular database such as KEGG or PubChem. As a preprocessing step, one needs to generate the true fingerprint vectors of each molecule in the database.

In matching the predicted fingerprints to the observed ones in the database, it is sensible to give more weight to fingerprints that can be predicted reliably from the mass spectrum. To implement that idea, FingerID uses a probabilistic model that exploits the cross-validation accuracies 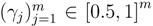 of the fingerprints as the reliability scores. Given the reliability scores and the predicted fingerprints ŷ, the model assigns the Poisson-binomial probability for the fingerprint vector y as follows:

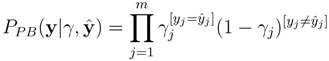

The above can be interpreted as measuring the likelihood of a fingerprint y to be the source generating the observations ŷ. For a molecule *M*, the probability of its fingerprint vector y(*M*) gives its score used in candidate retrieval:

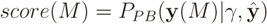
(3)


It is useful to reduce the number of candidates by filtering the molecular database by the exact mass of the molecule. If the mass is not known, it is estimated from the MS2 or MS1 spectrum. A a small search window is set [*µ_M_* − Δ, *µ_M_* + Δ] around the estimated mass *µ_M_* of the unknown molecule, and the records in the molecular database that exceed the allowed mass difference are filtered out. The size of the candidate set is obviously dependent on the width of the search window. A smaller width gives a smaller candidate set but has a higher risk to miss the true molecule, if the exact mass was estimated incorrectly. Based on the fingerprint scores in Equation (3), a ranked list is generated for the molecules within the mass window.

### 2.4. FingerID Web Server

**Figure 2:**
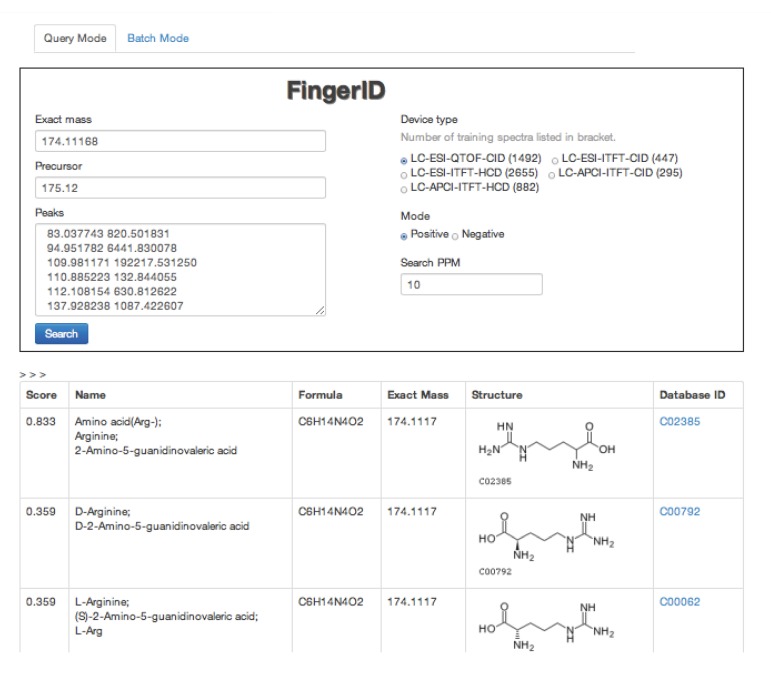
A screen-shot of FingerID web server.

The FingerID web server [19] was initially built for tackling the CASMI challenges. The server provides easy access to user who wishes to try the machine learning framework for metabolite identification. The underlying database on the server is KEGG. A screen-shot is shown in Figure 2.

The FingerID web server has two modes. In the query mode, the user can submit an http form to the server with the information related to the unknown molecule. The server will send back the result in a table. In the batch mode, the user can write the queries in pre-defined format files and compress them in a package. Then the user can upload the package to the server and the server will send back a package of results to the user.

Both modes share the same set of input search parameters. *Exact mass* is used in the beginning of database matching. *Search PPM* specifies the width of the mass window. It should be adjusted according to the assumed precision of the exact mass. *Precursor* is required for the *mloss* feature. *Peaks* are simply the list of masses and intensities of the MS/MS. *Device type* determines which trained model are used for molecular fingerprint prediction. The user should set the *Device type* according to his own mass spectrometer type. *Mode* tells the ionization mode of the mass spectrometer and it is useful in both estimating the exact mass and aligning the peaks.

### 2.5. FingerID Software Distribution

When using the FingerID web server, the user can only use the trained model provided by the web server administrators. The FingerID package [13] allows the user to select his own training mass spectra to train the prediction models. The training spectra should be in MassBank [5] format. Model parameters and database search parameters are specified in a configuration file. Training process could be minutes to hours or even more, depending on the size of the training data. More detailed instructions can be found on sourceforge project home page^5^ and in the readme file of the package.

## 3. Materials and Methods

### 3.1. CASMI Challenge Data

The 2012 CASMI contest [16] had four categories. Categories 1 and 2 are for high-resolution LC/MS data coupled with MS/MS while Categories 3 and 4 are for nominal mass GC/MS data. Categories 1 and 3 concern the identification of the chemical formula and Categories 2 and 4 concern the identification of the molecular structure. FingerID utilizes MS/MS data, therefore, only the Categories 1 and 2 are relevant.

All the challenge molecules are measured by two devices: Bruker micrOTOF-Q and LTQ-Orbitrap in which APCI ionization and ESI ionization are both used. Some analytes are better ionized by APCI and even in the cases that ESI and APCI deliver comparable ionization efficiency, the analytes respond differently [20]. In addition, for the LTQ-Orbitrap, both collision-induced dissociation (CID) and higher energy collisional dissociation (HCD) appear in the challenges and the resulting spectra for the same molecules are quite different.

For those CASMI challenges not explicitly indicating the precursor mass, it is assumed to be the molecular ion with some adducts, usually [M+H]^+^. With MS1 data at hand, estimating the exact mass of the unknown molecule is usually straightforward. Most MS1 spectra in the CASMI challenges have clear isotopic distributions. If the spectrum is measured in positive mode, the exact mass of the molecule can be computed as the most abundant peak in MS1 minus the mass of the proton. The most abundant peak was taken as the precursor if it is not given. However, positive mode does not necessarily imply [M+H]^+^. Other adducts such as [M+2H]^+^ and [M+Na]^+^ are also possible. Furthermore, noise and measurement error may lead to the difference larger or smaller than the mass of the proton.

### 3.2. Mass Spectral Training Data

Due to the differences in the CASMI challenges we decided to train different fingerprint prediction models for the different setups. In particular, we define three variables for our datasets: instrument type, ionization type and fragmentation method. According to these variables, the challenge data can be categorized to 5 models, which are (1) LC-APCI-ITFT-CID; (2) LC-APCI-ITFT-HCD; (3) LC-ESI-ITFT-CID; (4) LC-ESI-ITFT-HCD and (5) LC-ESI-QTOF-CID.

The summary of training data obtained from MassBank is listed in the first part (MS2) of Table 1. The molecules in Model (1) are a subset of Model (2), the molecules in Model (3) are a subset of Model (4). The molecules in Model (5) are quite different, with at most 5 molecules overlapping with the other models. Models (1) and (2) have 5 molecules also in Models (3) and (4).

**Table 1:** The training datasets statistics. The number of molecules is smaller than the number of spectra because of the existence of mass spectra of the same molecules measured in different collision energies.

MS type	Instrument type	No. of spectra	No. of molecules	Fingerprints
MS2	(1) LC-APCI-ITFT-CID	295	65	179
	(2) LC-APCI-ITFT-HCD	882	86	181
	(3) LC-ESI-ITFT-CID	447	224	281
	(4) LC-ESI-ITFT-HCD	2655	225	281
	(5) LC-ESI-QTOF-CID	1027	523	290
MS1	LC-ESI-ITFT	41	41	-
	LC-ESI-QTOF	62	62	-

An auxiliary dataset named *QqQ*, which contains spectra of collision energies of 10 eV (491 molecules), 20 eV (502 molecules), 30 eV (502 molecules), 40 eV (490 molecules) and 50 eV (449 molecules), is used to analyze the effect of different collision energies on the fingerprint prediction.

### 3.3. Molecular Fingerprints

OpenBabel [21] was used to generate molecular fingerprints, FP3 (55 bits), FP4 (307 bits) and MACCS (166 bits), all together 528 bits. However, in each dataset many of the fingerprints are either present in all molecules (+1) or absent (−1), which means they provide no information for training. We removed these ineffective fingerprints from each dataset. The number of the remaining effective fingerprints are shown in Table 1.

### 3.4. Molecular Databases

For the CASMI challenges, the KEGG [14] compound database was usedas the underlying molecular database, which contained 11,657 molecules. After the CASMI submission, the experiments with PubChem, which contains more than 30 million molecules, as the molecular database in place of KEGG were conducted.

### 3.5. SVM Model Training and Evaluation

Multiple spectra related to a single molecule are a potential source of bias in a cross-validation setting, if some of the spectra end up in the testing fold while others are present in the training fold. In such cases, the cross-validation accuracy becomes artificially high. To avoid this problem, the following stratified cross-validation scheme was used in training the models: all spectra related to a particular molecule were confined to the same cross-validation fold. Thus, in each trained model, either all spectra of a given molecule were present in the training data and none in the testing, or *vice versa*.

The margin softness parameter “*C*” for SVM is chosen from the list of [2^−5^, 2^−4^,..., 2^10^] independently for every fingerprint and training fold.

## 4. Results and Discussion

This section begins by reporting and analyzing the CASMI challenge results. Next, the different design choices in our approach and their effect on the metabolite identification performance are examnied. Finally, the extensions and improvements to FingerID that were not used in the CASMI challenge are presented.

### 4.1. CASMI Challenge Results

The FingerID results submitted to the CASMI contest is shown in Table 2. The model that was used for each challenge to predict fingerprints is also included in this table. Some challenges can map to several models, such as Challenge 13 where both CID and HCD data are available. In this case, the model with better cross validation performance was used, which is Model (4) LC-ESI-ITFT-HCD.

The results for the molecule identification (Category 2) were computed first, and the chemical formula identification is directly taken from molecule identification. As a result, it is more meaningful to discuss the results of Category 2. Expectedly, molecule identification proves to be a harder problem than chemical formula prediction. As an overall finding, most of our failures in the challenges are due to the limited molecular database used for retrieval: the molecules in challenges 3,4,10,12–17 were not in our version of KEGG. Exception is Challenge 11 in which the exact mass was estimated incorrectly.

**Figure 3:**
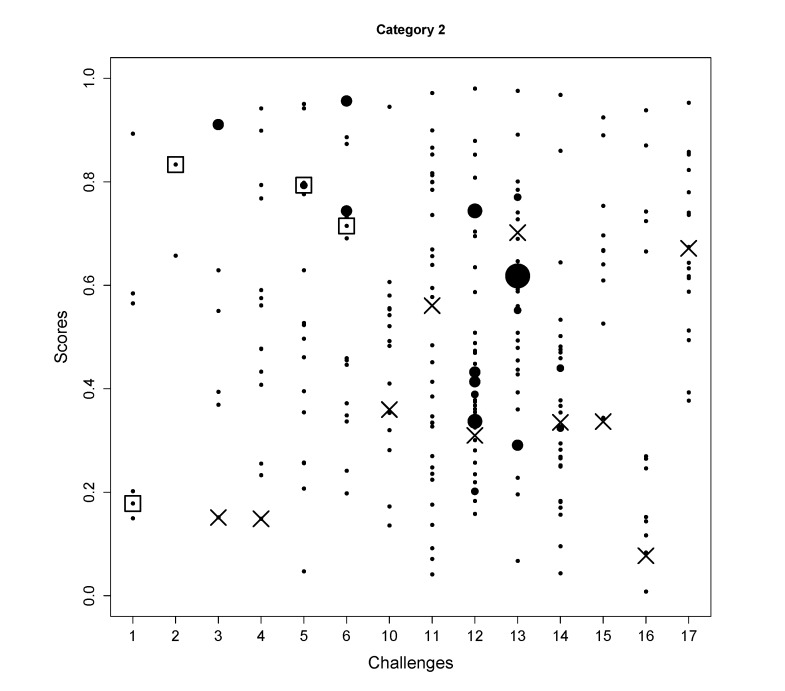
The scores of all candidates (y-axis) for the challenges (x-axis) are shown in black dots where the size indicates the number of candidates sharing the same scores. The correct solutions are indicated with a square. For the challenges where the correct molecules were not in the database, the hypothetical rankings (assuming the molecules were in the database) of the true molecules are shown by the cross markers.

The distributions of the scores for all the candidates in 14 challenges in Category 2 are shown in Figure 3. One observation is that the score distributions are quite flat, indicating good discriminability of FingerID. Below, the results of those challenges where the correct molecules were in the KEGG are discussed individually.

**Table 2:** Absolute ranks of the correct molecules in the solution lists for the challenges in Category 1 and 2 and the number of candidates. Challenges where the correct molecule is not in the result list are marked with “-” instead. The model used for each challenge to predict the fingerprints is listed in the Model row. The proportions of wrongly predicted molecular fingerprints are shown in the last row.

Challenge	1	2	3	4	5	6	10	11	12	13	14	15	16	17
Model	(5)	(5)	(5)	(5)	(5)	(5)	(1)	(1)	(1)	(4)	(2)	(2)	(2)	(4)
Category 1 rank # candidates	**4**	**1**	**-**	**3**	**4**	**4**	**1**	**-**	**-**	**-**	**1**	**5**	**-**	**-**
	5	4	6	12	11	15	11	15	21	15	12	6	12	11
Category 2 rank # candidates	**5**	**1**	**-**	**-**	**5**	**11**	**-**	**-**	**-**	**-**	**-**	**-**	**-**	**-**
	6	6	8	14	17	20	15	32	47	38	28	10	13	18
Wrong FP(%)	25	33	32	33	30	34	23	25	32	19	21	31	41	24

- In Challenge 1, the correct molecule was in the fifth position out of six. The score of the correct molecule was rather low; the high rank is probably a consequence of KEGG not having many molecules with a similar molecular weight, rather than good fingerprint prediction.
- In Challenge 2, the search ppm was set to 200 and the correct solution was obtained despite the 30 ppm error in the original challenge data. In the version of KEGG, only three entries have the mass around 592.1792 within 200 ppm and only two of them had molecular fingerprint generated using OpenBabel. Thus, the identification is simply choosing one from the two and the FingerID made the right choice. Incidentally, after correcting the 30 ppm error, FingerID still ranked the true molecule at the top, which surpassed other CASMI participants.
- In Challenge 5, the correct molecule ranks fifth and had the same confidence score as the fourth one. If the CASMI organizers took the rank of the score as criterion, this would have been the winning entry for this challenge.
- In Challenge 6, half of the candidates had better scores than the correct one, which means the molecular fingerprint prediction was not perfect. However, comparing the absolute rank to the other participants, this was sufficient to win this challenge.
- In Challenge 11, the highest intensity peak in MS1 is not the molecule with an adduct. Thus, the exact mass of the molecule was estimated incorrectly.

The question of how the challenge molecules differ from the training molecules and molecules in the database is answered by comparing the fingerprints , as shown in Appendix A. A simple similarity function between fingerprints is defined and histograms of the similarity scores are presented.

### 4.2. Evaluation of the FingerID Framework in the CASMI Contest

Here we study the effects of different aspects and design choices in FingerID that explain the CASMI results and point directions for future improvement. The robustness of the fingerprint prediction model and the prediction of the exact mass are first studied, followed by a discussion of how to utilize mass spectra measured by different collision energies. Finally, the uniqueness of fingerprints is also explored.

#### 4.2.1. Effect of Training Set Size on Fingerprint Prediction Reliability

**Figure 4:**
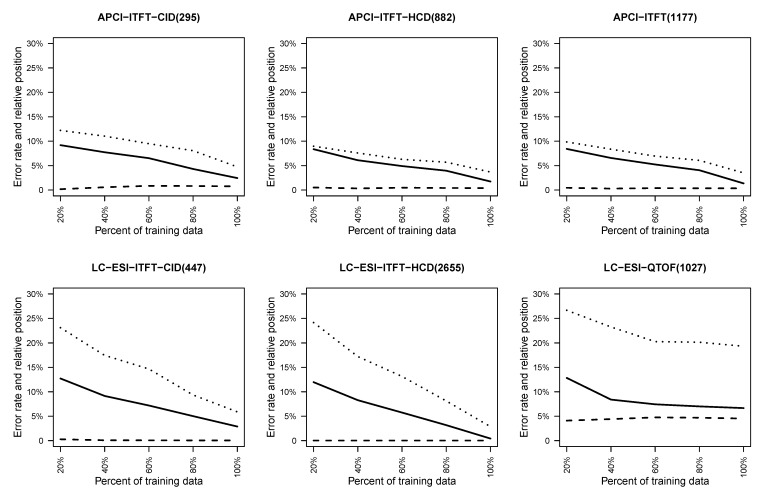
This figure shows for different size subsamples the average cross-validation test error (solid lines) and training error (dashed lines) over all fingerprints, and the average relative rank (dotted lines) of the correct molecule in the list of retrieved candidates.

To understand how the size of training set affects the fingerprint prediction accuracy, subsamples of the data of gradually increasing size were generated and 10-fold stratified cross-validation (see Section 3.5) on each subsample was conducted. Each time one fold was picked as testing data and 20%, 40%, 60%, 80% and all of the remaining data were extracted as training data. The resulting curves for cross-validation and training error are shown in Figure 4, together with the relative rank of the retrieved molecule. In these experiments, fingerprints for which the majority class accounts for at most 80% are included.

A general trend in Figure 4 is that with more data to train, lower testing error and better relative rank of the correct molecule were observed. An exception is the LC-ESI-QTOF dataset where it seems the performance after training with 60% of the data could not be improved much. For the LC-ESI-ITFT-HCD, the cross validation testing error approaches zero rapidly. The APCI-ITFT dataset, which is the combination of the APCI-ITFT-CID and the APCI-ITFT-HCD, shows that the merging produces slightly better performance than simply using the APCI-ITFT-CID data but slightly worse result then just using the APCI-ITFT-HCD data.

In summary, most of the datasets seem to be large enough to give good average prediction quality for fingerprints. In addition, the metabolite identification performance is seen to correlate with the fingerprint prediction error in a clear way. However, the datasets are too small to represent the whole metabolite space in balanced manner. Hence, the good results within each dataset may not translate to good metabolite identification results outside the region of metabolite space covered by the training data.

#### 4.2.2. Quality of Exact Mass Prediction

**Figure 5:**
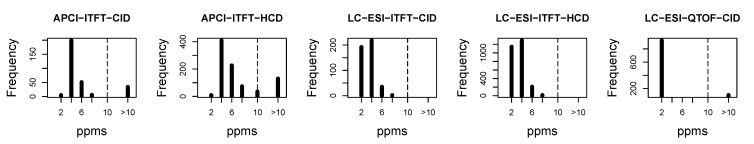
Histogram of the difference between derived mass and exact mass, measured in ppm, intercepted at 10. The difference less than 10 ppm accounts for 88%, 85%, 100%, 100%, 90% of the difference between derived mass and exact mass for five datasets respectively.

To understand how accurate the approach of deriving the exact mass from the spectra of the unknown molecules, the difference (measured in ppm) between the exact mass and the predicted exact mass for our training datasets was compared, as shown in Figure 5.

Figure 5 shows in most cases, the difference between the predicted and the real exact mass of the molecule is less than 10 ppm, which indicates the suitable mass window width. For the CASMI contest, the mass window width was set to range from 200 ppm to 500 ppm even though in hindsight our data would have supported a much narrower search window. In the APCI-ITFT-CID, APCI-ITFT-HCD and LC-ESI-QTOF-CID datasets, there are some outliers where the exact mass prediction is off by huge amount (1000s of ppms), which need to be investigated in the future.

#### 4.2.3. Effect of Using Multiple Collision Energies

As observed from Table 1, many spectra of the same molecule exist in the training dataset. This is a result of different collision energies used in the measurement, which leads to different fragmentations of the precursor ions.

**Figure 6:**
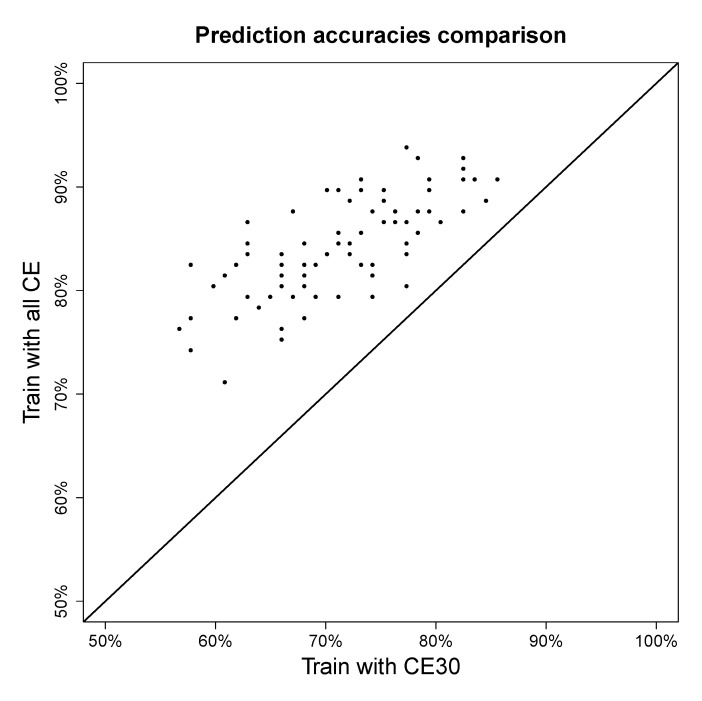
Fingerprint prediction accuracy on *QqQ* data with single collision energy data versus mixed collision energy data. The x-axis shows the accuracies of training model only using collision energy 30 eV and the y-axis shows the accuracies of training model using all available collision energies.

To test if mixing the different collision energies in training data has a positive or negative effect on fingerprint prediction, one fifth of the molecules measured in collision 30 eV in the *QqQ* dataset were taken as fixed testing data and the rest of the dataset was used for training in two setups: training with 30 eV only and training with all collision energies. The result is shown in a scatter plot in Figure 6. In this experiment, only fingerprints for which the majority class accounts for at most 80% of the data were considered, as achieving high predictive accuracy for those is more challenging than the more biased fingerprints.

Figure 6 shows that even though the resulting spectra of different collision energies are not the same, integrating them as one model always improves prediction accuracy. Combining spectra from different collision energies instead of building a model for every single collision energy reduces model complexities and gains prediction accuracy.

#### 4.2.4. Degree of Uniqueness of Fingerprints

**Figure 7:**
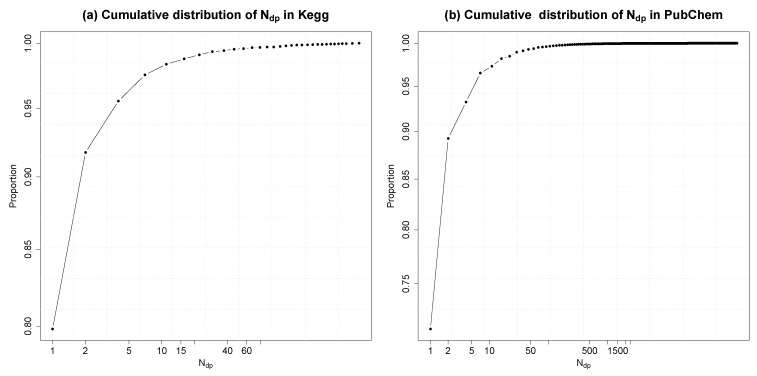
Cumulative distribution of *N_dp_*, which is the number of molecules having the *N_dp_* duplicates or less. Both x-axis and y-axis are in log scale.

Molecular fingerprints describe the selected properties of a molecule in the form of a bit vector. In FingerID, the molecular fingerprint is an intermediate representation for identifying candidate molecules. As the ranking of the candidates is based on the fingerprint vector and the mass of the molecule, fingerprint vectors that are shared by large number of molecules cause the candidate lists to grow in size, which is not desirable. In this experiment, for each different fingerprint configuration in KEGG and PubChem, respectively, the duplicates in the database were counted and denoted as *N*_*dp*_. We show the cumulative distribution of *N*_*dp*_ (in log scale) in Figure 7.

Fingerprints with *N*_*dp*_ = 1 are unique in the database. In the versions of KEGG and PubChem databases used, we have 9,399 and 18,043,141 different fingerprint configurations, respectively. In KEGG (resp. PubChem), among them, 7,454 (resp. 12,829,187) fingerprint configurations are unique, and 1,114 (resp. 3,282,098) fingerprint configurations have one duplicate molecule. The highest number of duplicates in KEGG is 86 and 2,501 in PubChem. In terms of molecules, only 64% of the molecules in KEGG and 43% in PubChem have unique fingerprints.

For molecule identification, the harmful effect of the duplicate fingerprints existing in the database is partly diluted by considering the masses of the molecules: the percentage of molecules with unique fingerprints and unique mass are 75% and 73% in KEGG and PubChem, respectively.

### 4.3. Extensions

In the following, the extensions of FingerID that were not used in the CASMI contest are examined, namely using isotopic distribution information from MS1 spectrum and the use of PubChem as the molecular database instead of KEGG.

#### 4.3.1. Isotopic Distribution Matching

Each chemical element can have several isotopes that share the same protons and electrons but different number of neutrons. The isotopes occur in nature in certain abundances. For example, carbon has two stable isotopes ^12^C and ^13^C with abundance of 98.890% and 1.110%.

For an element *E* with *r* isotope types, a molecule consisting of *l* atoms of that element has 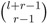 different isotopologues caused by that element [22]. All of the isotopologues also have distinct abundances, which can be derived by applying multinomial probability over the isotope abundances [23,24]. Consequently, the theoretical mass spectrum that arises from the set of isotopologues can be simulated and compared with the observed spectrum. This information is more informative for metabolite identification than using the mass alone. Many methods and tools have been published for this purpose [25,26,27].

In the CASMI challenges, MS1 data were also given and most of them contain isotopic distributions. This allows us to rank the candidates based on matching between observed isotopic distribution and simulated isotopic distributions from the chemical formulas. For the matching score, the probability product kernel function (2) was used.

The fingerprint based score (3) was fused with the isotopic distribution based score to obtain the final ranking. Two rank aggregation methods are investigated: taking the average rank or minimum rank as the combined ranking. Some molecules may have the same chemical formulas and thus they receive the same isotopic distribution matching scores. Another slightly more complicated method is ranking the candidates by the matching isotopic distribution score first and for those candidates having a tie, reranking them by the fingerprint based scores.

MS1 data was used for LC-ESI-ITFT and LC-ESI-QTOF datasets as shown in the last two rows of Table 1. Compared with MS2 data, only a few molecules have the MS1 data in the database. FingerID was trained with the MS2 data and the MS1 data was used for isotopic distribution matching. Then the combining method mentioned above was applied to merge two ranked list.

**Figure 8:**
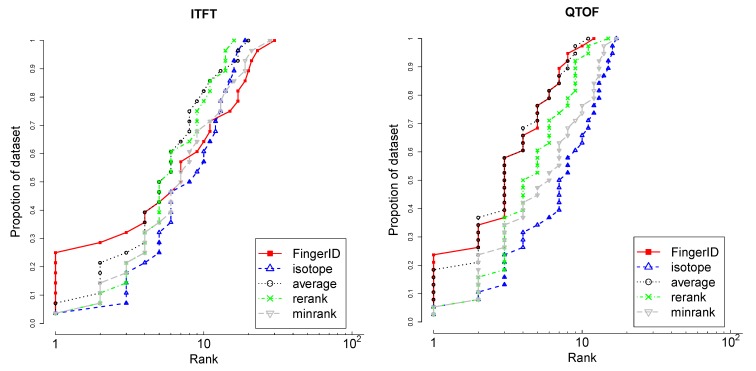
The rank distribution of metabolites using FingerID alone, isotope matching alone and the different rank aggregation schemes; average rank, minrank and rerank. The higher the curve, the larger the proportion of highly ranked molecules. The x-axis is in log scale.

Figure 8 shows that FingerID alone achieves top 1 rank for the largest fraction of the data for both datasets. It is also shared best with average rank aggregation on the whole of QTOF data. On ITFT data, FingerID alone is the best until top 5 rank but fades beyond the competing methods in ranking the tail of the data. Reranking with FingerID the candidates with matching isotopic distributions is a better approach than minimum rank. As the CASMI challenges used the absolute rank of the correct molecule as the goodness criterion, the FingerID alone was used to solve the challenges.

#### 4.3.2. Using PubChem as the Molecular Database

In the CASMI contest, KEGG was used as the underlying database to search candidates. As many of the challenge molecules were not recorded in KEGG, the method failed to identify those molecules correctly. After the challenge deadline, PubChem [15] was investigated as the alternative source of candidate molecules. As PubChem contains more than 30 million compounds, several orders of magnitude larger than KEGG, the recall of molecules is improved. At the same time, however, the number of candidates within a mass window increases, making it harder to rank the correct molecules towards the top. Table 3 shows the result for Category 2 when using PubChem as the underlying molecular database.

**Table 3:** Retrieval of candidate molecules from PubChem. The top part of the table corresponds to the CASMI setup except for replacing KEGG with PubChem. The middle part shows the results with 10 ppm mass window with PubChem. The bottom rows depict the best possible results that could be obtained if the optimal mass window width was known.

Challenge	1	2	3	4	5	6	10	11	12	13	14	15	16	17
Mass window (ppm)	300	200	500	300	300	300	500	500	500	500	600	500	300	500
Candidates	55,242	9,931	87,514	75,205	75,320	115,639	26,701	-	-	69,708	10,390	18,669	97,567	19,718
Cat 2 rank	70	2	355	12,699	4,050	6,881	11	-	-	13,796	341	13,689	71,410	8,350
10 ppm rank	25	-	5	2,225	1,343	1,048	7	-	-	166	57	1,815	2,416	231
Ideal ppm	2.34	50	3.90	9.38	1.17	9.38	0.98	-	-	0.98	9.38	7.81	0.98	1.95
Ideal rank	23	2	4	2,225	1,337	1,048	7	-	-	15	57	1,780	775	198

The top three rows show the results obtained when searching PubChem using the same mass window width as was used in the CASMI challenges. It can be observed that for most of the challenges the correct molecules are among the retrieved candidates, with the exception of Challenge 11, in which the precursor mass was estimated incorrectly, and Challenge 12, where the molecule is not found in the version of PubChem. The candidate lists are quite long and the absolute ranks of the correct molecules are typically too low to allow manual checking by a human expert. For example, the correct molecule in Challenge 3 is ranked as 355 out of 87,514, which corresponds to top 0.4% of the retrieved candidates.

The fourth row of the Table 3 shows the effect of choosing a much smaller mass window of 10 ppm, based on the statistics in Figure 5, which suggest that larger errors in exact mass prediction are relatively rare. This result shows remarkable improvement in ranking the challenge molecules. In fact, these could have won Challenges 3 and 10. Correspondingly, in Challenge 2 the correct candidate would have been pruned out due to the 30 ppm error in the mass.

In the last two rows of Table 3, the idealized case where the smallest ppm range is sought for that still keeps the correct molecule in the candidate list is inspected. *Ideal ppm* is defined as the smallest value for ppm that still allows the candidate list contain the correct molecule, and *ideal rank* is the corresponding rank that is achieved by using the ideal ppm mass window. It can be noted that ideal ranks are in many cases not much better than that achieved using the 10 ppm mass window.

## 5. Conclusions

The prediction of molecular fingerprints from tandem MS/MS using machine learning methods tackles the molecule identification problem in a brand new way. In this approach, observed MS/MS spectra are not directly compared with an MS spectral database or simulated MS/MS spectra. Instead, molecular fingerprints are predicted and then used to search a molecular database of choice. The CASMI contest results show this machine learning approach is competitive with other current methods.

This approach is modular in that the support vector machine used in this paper could be changed to any other machine learning approach. The set of fingerprints generated by OpenBabel can be replaced by others such as PubChem fingerprints. Finally, the molecular database used to retrieve candidate molecules can be changed flexibly.

The presented approach still requires further development. The first issue is that the prediction is highly dependent on the selection of the training dataset. Using merely a few hundred molecules as training data cannot represent the whole relevant chemical space. Hence, some testing molecules that are beyond the learned model domain may be encountered. However, as the results so far suggest, relatively good performance can already be achieved with moderate training sets of a few hundred molecules. Thus, extending the approach to new regions of metabolite space via generating spectral training data for the sparsely populated regions should not prove an insurmountable challenge.

The molecular fingerprint prediction also incurs the necessity for studying the properties and structures of the molecular fingerprints. Appendix A shows lots of molecules sharing the same fingerprints in the KEGG and PubChem, even through 528 bits OpenBabel substructure fingerprints can theoretically produce 2^528^ fingerprint configurations. Simply changing OpenBabel fingerprints to a larger set of fingerprints (e.g., PubChem fingerprints) can reduce some duplicates, but more detailed research within the set of fingerprints is required.

There are several ways to refine the set of fingerprints and their predictions. First, modeling the dependencies among the fingerprints could improve predictive accuracy, such as learning a Bayesian network of fingerprints and using the learned dependency graph as the input for structured learning. Second, weighting the fingerprints by the ability to differ molecules could help the ranking of candidates.

Choosing the molecular database for candidate retrieval is the final crucial component. A small database will produce shorter candidate lists but with high chance the correct molecule is not in the list, unless the molecules to be identified match the database very well. A much larger database such as PubChem includes the correct molecule in many cases but may produce a prohibitively large candidate lists. Using a small enough mass window can alleviate this problem to significant degree.

There are several further directions for improving the metabolite identification framework. First, FingerID does not use information of plausible fragmentation trees, which are effectively used in several competing systems. Second, using domain specific information about the kinds of molecules that are more plausible than others could improve the framework. Finally, the molecular database can be discarded if combinatorial algorithms are developed to reconstruct the molecule structure given the predicted molecular fingerprints.

## Figures and Tables

**Figure A1:**
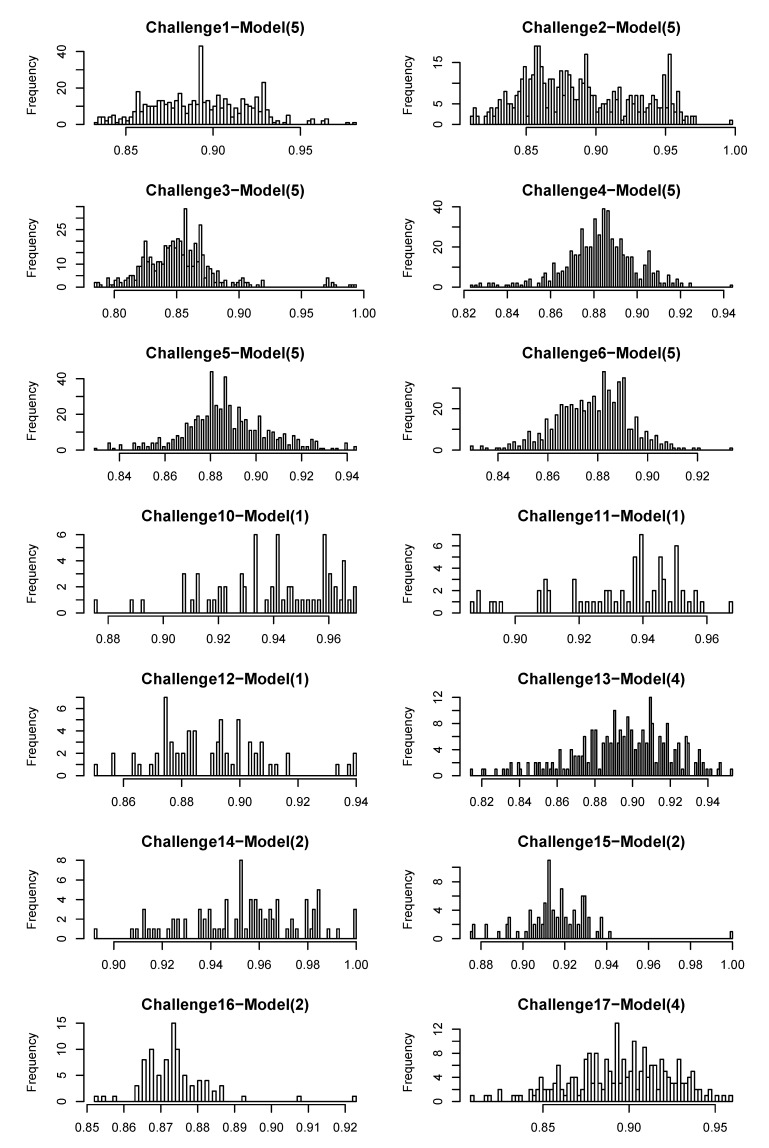
Distribution of similarity scores between challenge molecules and training data (used to predict the challenge).

**Figure A2:**
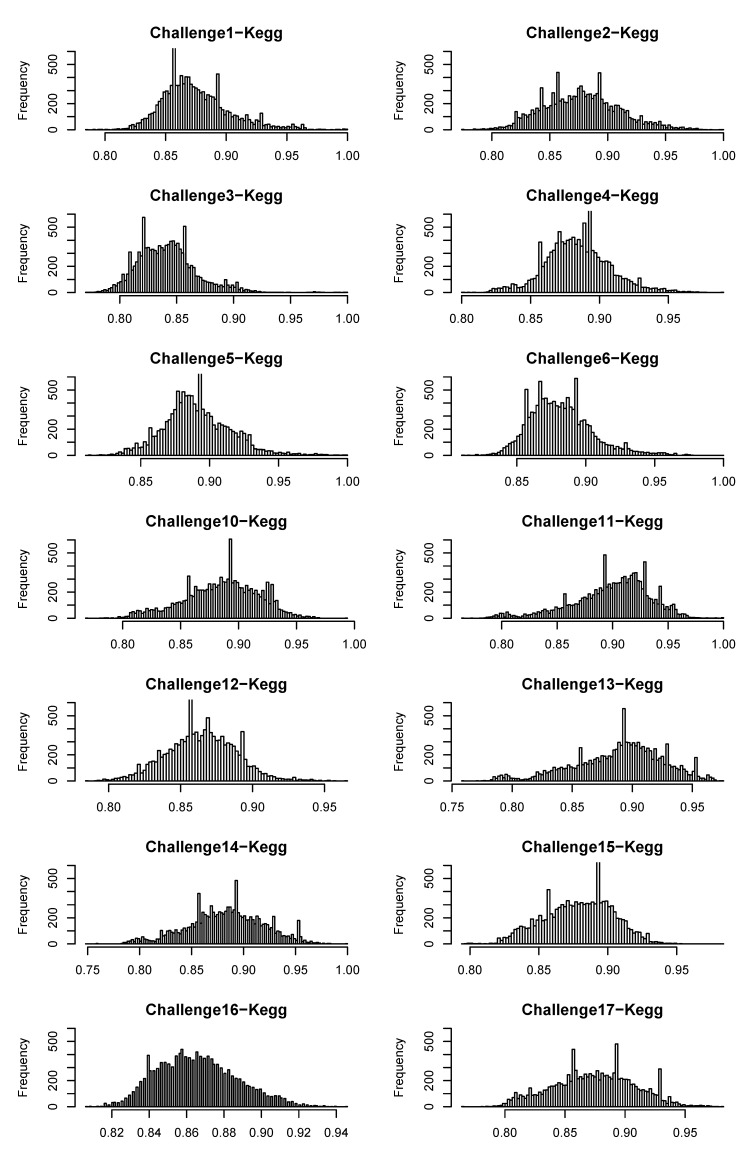
Distribution of similarity scores between challenge molecules and molecules in the KEGG compound database.

## Appendix

### Appendix A

This appendix contains the definition of similarity between two fingerprints and similarity distribution between CASMI challenge molecules in Category 1 & 2 and training molecules (Figure A1) and molecules in KEGG compound database (Figure A2).

Given two vectors of *m* molecular fingerprints,  and , the similarity between them, if no prior knowledge exists on the weight of each individual fingerprint, is defined by:

By this definition, two same fingerprints give similarity score of 1 while two totally different fingerprints give similarity score of 0.
